# Supplementary material for: Ciliary tip actin dynamics regulate photoreceptor outer segment integrity
Source: Nat Commun. 2024 May 21;15:4316. doi: 10.1038/s41467-024-48639-w (PMC11109262; doi:10.1038/s41467-024-48639-w)
Supplement: Supplementary file 1 — Supplementary Information [file 41467_2024_48639_MOESM1_ESM.pdf]

Supplementary Information

Ciliary tip actin dynamics regulate photoreceptor outer segment integrity

Roly Megaw,[1,2]* Abigail Moye,[3] Zhixian Zhang,[3] Fay Newton,[1] Fraser McPhie,[1] Laura C. Murphy,[1] Lisa McKie,[1] Feng He,[3] Melissa K. Jungnickel,[1] Alex von Kriegsheim,[4] Peter Tennant,[1] Chloe Brotherton,[1] Christine Gurniak,[5] Alecia K. Gross,[6] Laura M. Machesky,[7,8] Theodore Wensel,[3] Pleasantine Mill.[1]

[1] MRC Human Genetics Unit, MRC Institute of Genetics & Cancer, University of Edinburgh, Western General Hospital, Edinburgh, UK, EH4 2XU.

[2] Princess Alexandra Eye Pavilion, NHS Lothian, Edinburgh, UK, EH3 9HA.

[3] Verna and Marrs McLean Department of Biochemistry and Molecular Pharmacology, Baylor College of Medicine, Houston, Texas, USA, 77030.

[4] Edinburgh Cancer Research United Kingdom Centre, Institute of Genetics and Cancer, University of Edinburgh, Edinburgh, UK, EH4 2XU.

[5] Institute fur Genetik, Universitat Bonn, Karlrobert-Kreiten-Strasse, 53115, Bonn, Germany.

[6] University of Alabama at Birmingham, 2nd Ave South, Birmingham, Alabama, USA, 35294

[7] CRUK Scotland Institute, Switchback Road, Bearsden, Glasgow, UK, G61 1BD.

[8] Department of Biochemistry, University of Cambridge, CB1 7UY

* Corresponding author

Contents:

Supplementary Figures 1 - 10

Supplementary Tables 1 - 3

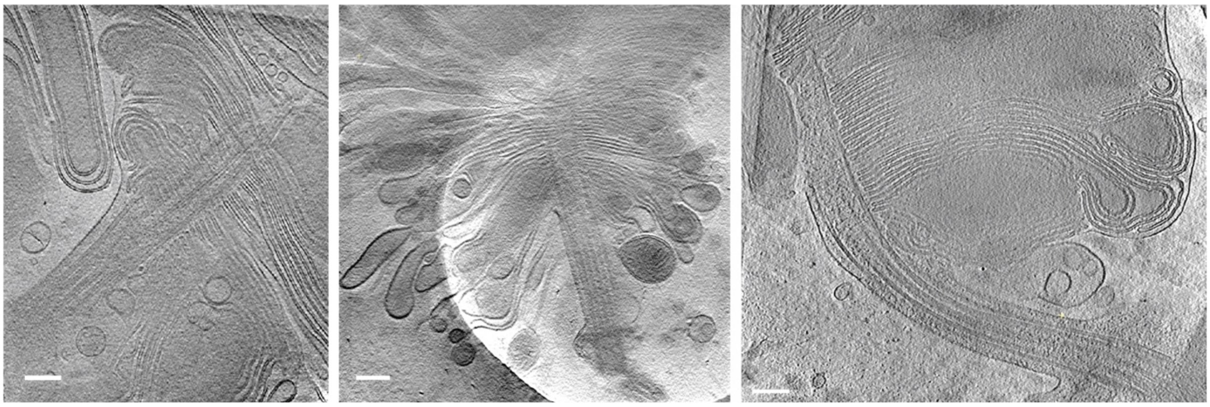

Supplementary Figure 1. Cryo-electron tomography allows imaging of photoreceptors at an ultrastructural level. Low magnification images demonstrate preservation of photoreceptor architecture. (CC = connecting cilium; OS = Outer Segment; Scale bar = 300 nm)

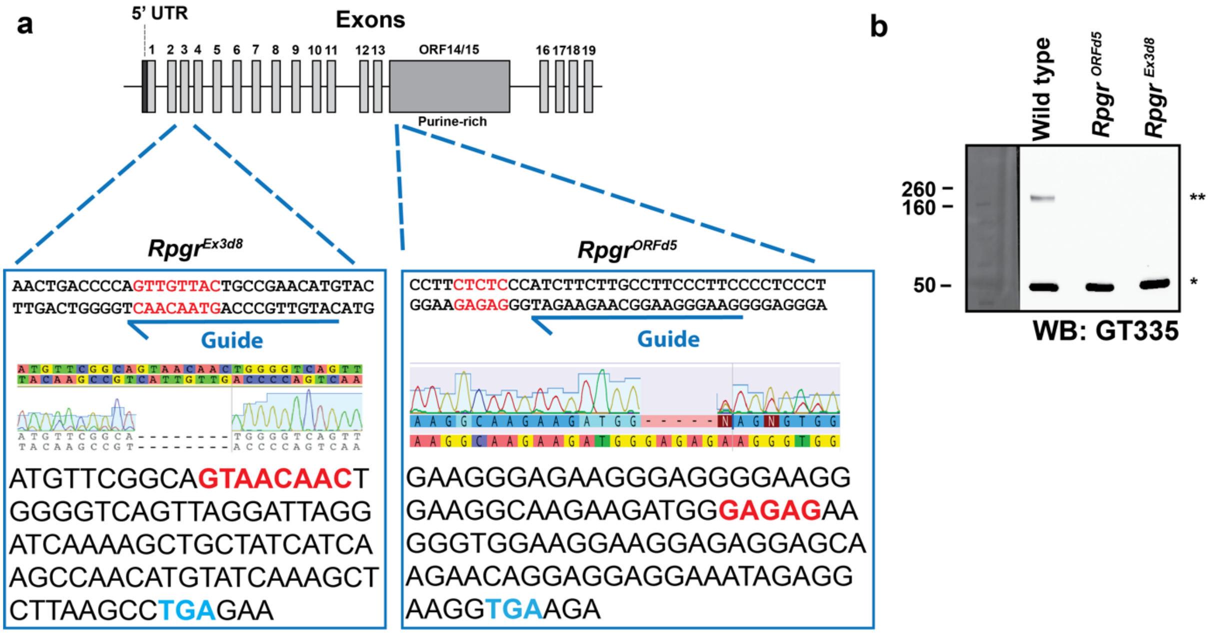

Supplementary Figure 2. Generation of novel murine *Rpgr* disease models. (a) Targeted pronuclear injection of CRISPR/Cas9 products using guide RNAs as indicated (see methods) induced deletions (red text) in Exon 3 (bottom left panel; termed $Rpgr^{Ex3d8}$) and the open reading frame (bottom right panel; termed $Rpgr^{ORFd5}$) of *Rpgr*, as detected by Sanger sequencing. This resulted in premature termination codons being shifted into frame (blue text). **(b)** Immunoblotting using a GT335 antibody detects the polyglutamylated, retinal specific isoform of Rpgr (**; see wild type lane)[34]. This band was lost in both the $Rpgr^{Ex3d8}$ and $Rpgr^{ORFd5}$models. The GT335 antibody also labels polyglutamylated tubulins (*), which serve as a loading control. This experiment was repeated twice with similar results.

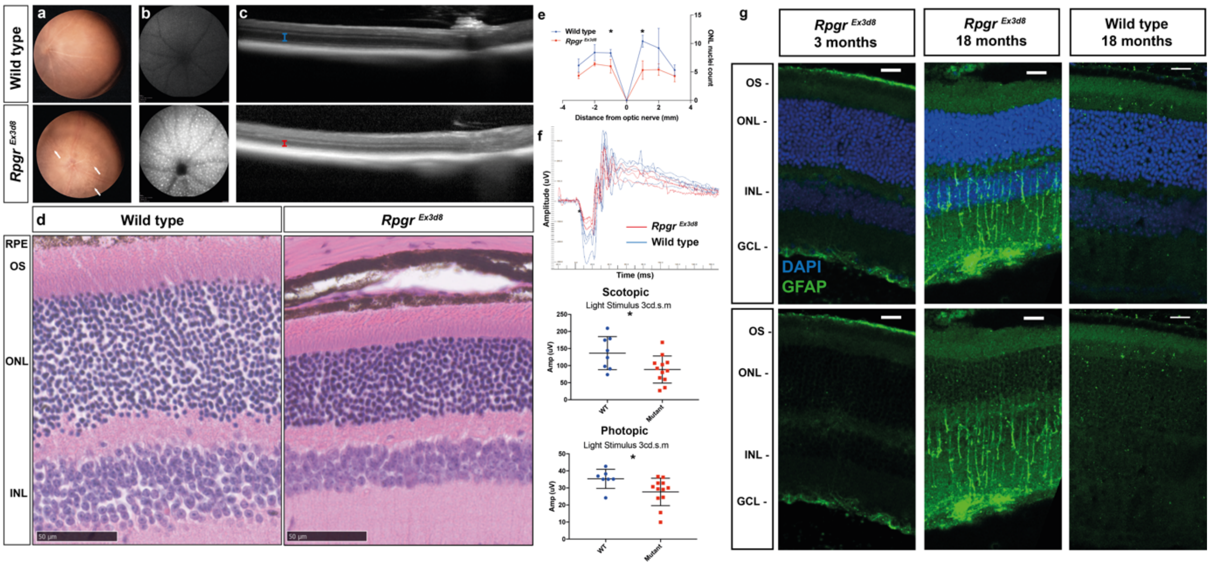

Supplementary Figure 3. A mouse model of *RPGR*/XLRP, *Rpgr*^{Ex3d8}, undergoes loss of photoreceptor function and structure. (a) Fundoscopy shows 18 month *Rpgr*^{Ex3d8} mouse develops white punctate retinal lesions (arrows). **(b)** Blue light autofluorescence shows accumulation of hyperautofluorescent material in *Rpgr*^{Ex3d8} retina. **(c)** *In vivo* optical coherence tomography shows ONL (photoreceptor) thinning in *Rpgr*^{Ex3d8} mice at 18 months (blue bracket in wild type; red bracket in mutant). (a-c; experiments repeated 3 times with similar results). **(d)** H&E staining of 18 month mice supports *in vivo* imaging, with thinning of ONL in *Rpgr*^{Ex3d8} mouse (RPE = retinal pigment epithelium; OS = outer segment; ONL = outer nuclear layer; INL = inner nuclear layer). **(e)** Spider plot demonstrates ONL loss (N = 3 animals per experimental group; * , p ≤ 0.05; * at -1 = 0.0399; * at 1 = 0.0103; two-tailed, unpaired t-test; data presented as mean values +/- SEM). **(f)** Electroretinogram shows loss of retinal function in *Rpgr*^{Ex3d8} mice; top panel shows representative scotopic tracings, with flattening of electronegative downflection ('a' wave; *) in *Rpgr*^{Ex3d8} mice, signalling loss of rod function; middle panel shows a-wave amplitudes in response to 3 candela dark-adapted stimulation; bottom panel shows amplitude of light-adapted photopic response, signalling loss of cone function (N = 7-12 animals per experimental group; *, p ≤ 0.05; photopic ERG * = 0.0402; scotopic ERG * = 0.0257; two-tailed, unpaired t-test; data presented as mean values +/- SEM). **(g)** Increased glial fibrillary acidic protein (GFAP) immunolabeling throughout radial length of Müller cells at 18 months in *Rpgr*^{Ex3d8} retinas indicating retinal stress. GFAP upregulation is not seen at 3 months, when *Rpgr*^{Ex3d8} mouse has already developed abnormal outer segment architecture (Figs. 2 a & b). (Scale bars; d = 1 mm; g = 20 µm). Source data provided as a Source Data file.

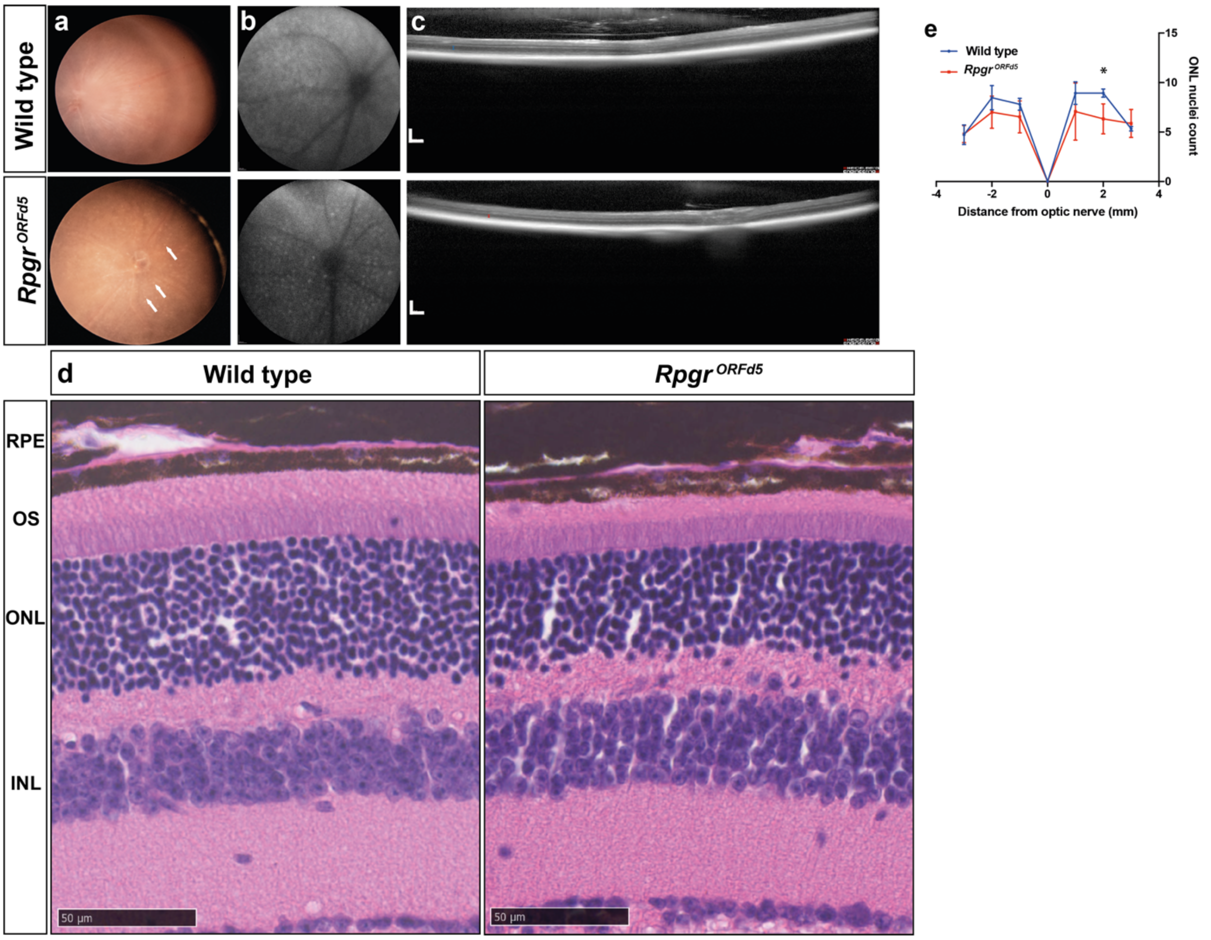

Supplementary Figure 4. A mouse model of *RPGR*/XLRP, *Rpgr*^{ORFd5}, undergoes loss of photoreceptor structure. (a) *Rpgr*^{ORFd5} mouse develops white punctate retinal lesions (white arrows) by fundoscopy. **(b)** Accumulation of hyperautofluorescent material in *Rpgr*^{ORFd5} retina by blue light autofluorescence. **(c)** *In vivo* optical coherence tomography shows outer nuclear layer (photoreceptor) thinning in *Rpgr*^{ORFd5} mice at 18 months of age (blue brackets in wild type; red brackets in mutant). (a-c; experiments were repeated 3 times with similar results). **(d)** Thinning of outer nuclear layer in *Rpgr*^{ORFd5} animal at 18 months by histology (H&E staining) (RPE = retinal pigment epithelium; OS = outer segment; ONL = outer nuclear layer; INL = inner nuclear layer;). **(e)** Quantification of outer nuclear layer (nuclei number) across the retina (N = 3 animals per experimental group; *, $p \leq 0.05$; * at 2 = 0.0456; two-tailed, unpaired t-test; data are presented as mean values +/- SEM). Scale bars; c = 200 µm; d = 1 mm). Source data are provided as a Source Data file.

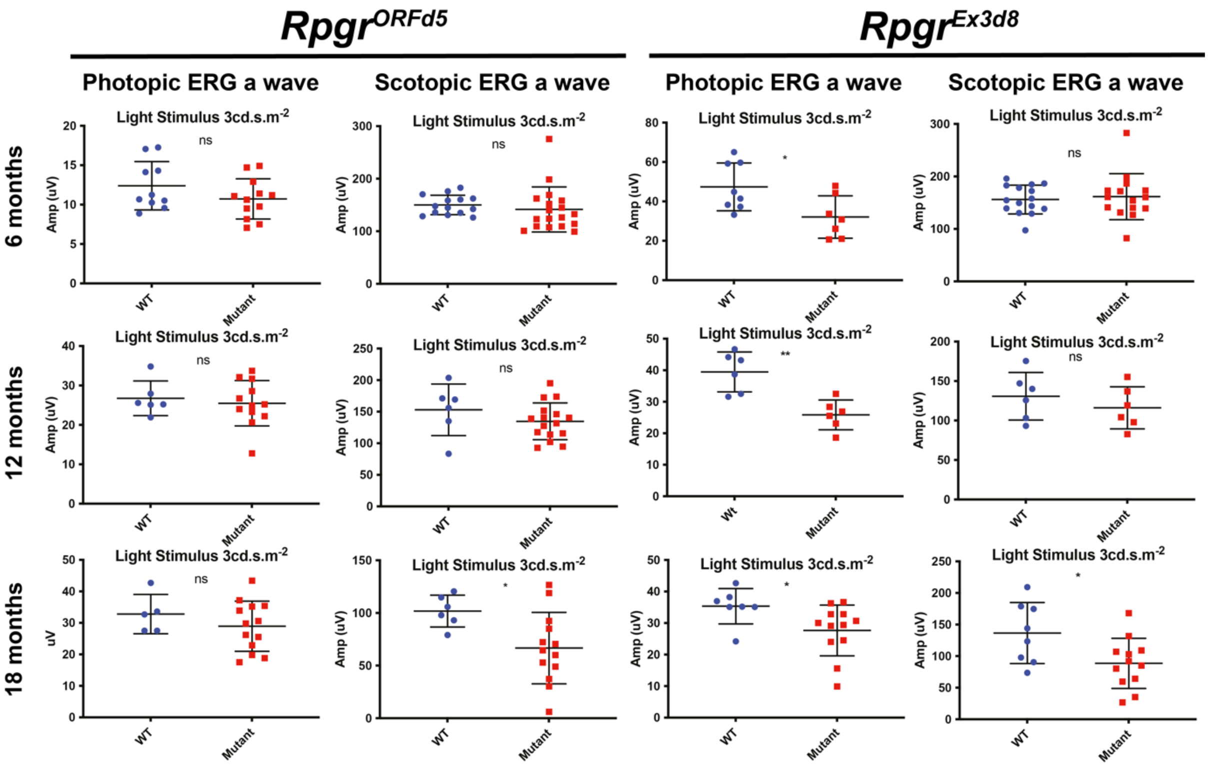

Supplementary Figure 5. Temporal ERG data shows retinal function declines over time in both mouse models of *Rpgr d*isease. Analysis of *Rpgr*^{ORFd5} (left 2 columns) and *Rpgr*^{Ex3d8} (right 2 columns) mice at 6, 12 and 18 months shows loss of rod photoreceptor (scotopic light stimulus 3cd.s.m^{-2}) function at 18 months in both strains. Analysis of cone photoreceptor (photopic light stimulus 3cd.s.m^{-2}) function shows early loss of cone function in *Rpgr*^{Ex3d8} mice but not in *Rpgr*^{ORFd5} mice (N = 7-12 per experimental group). *Rpgr*^{ORFd5} 18 month scotopic ERG * = 0.0276; *Rpgr*^{Ex3d8} 6 month scotopic ERG * = 0.0248; *Rpgr*^{Ex3d8} 12 month photopic ERG ** = 0.0017; *Rpgr*^{Ex3d8} 18 month photopic ERG * = 0.0402; *Rpgr*^{Ex3d8} 18 month scotopic ERG * = 0.0257. Two-tailed, unpaired t-test; data are presented as mean values +/- SEM. Source data are provided as a Source Data file.

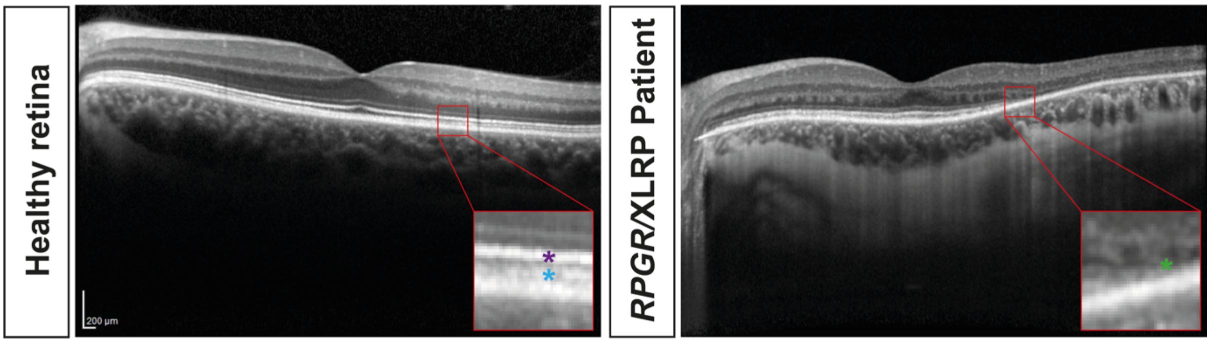

Supplementary Figure 6. Optical coherence tomography (OCT) shows loss of ellipsoid zone in *RPGR*/XLRP patient. OCT of healthy volunteer (left panel). Magnified insert shows the ellipsoid zone (magenta star) and interdigitating zone (cyan star) that together represent the photoreceptor outer segment. OCT of patient with *RPGR*/XLRP (right panel). Magnified insert shows loss of ellipsoid zone temporal to macula (extending centrally from right of scan up to green star).

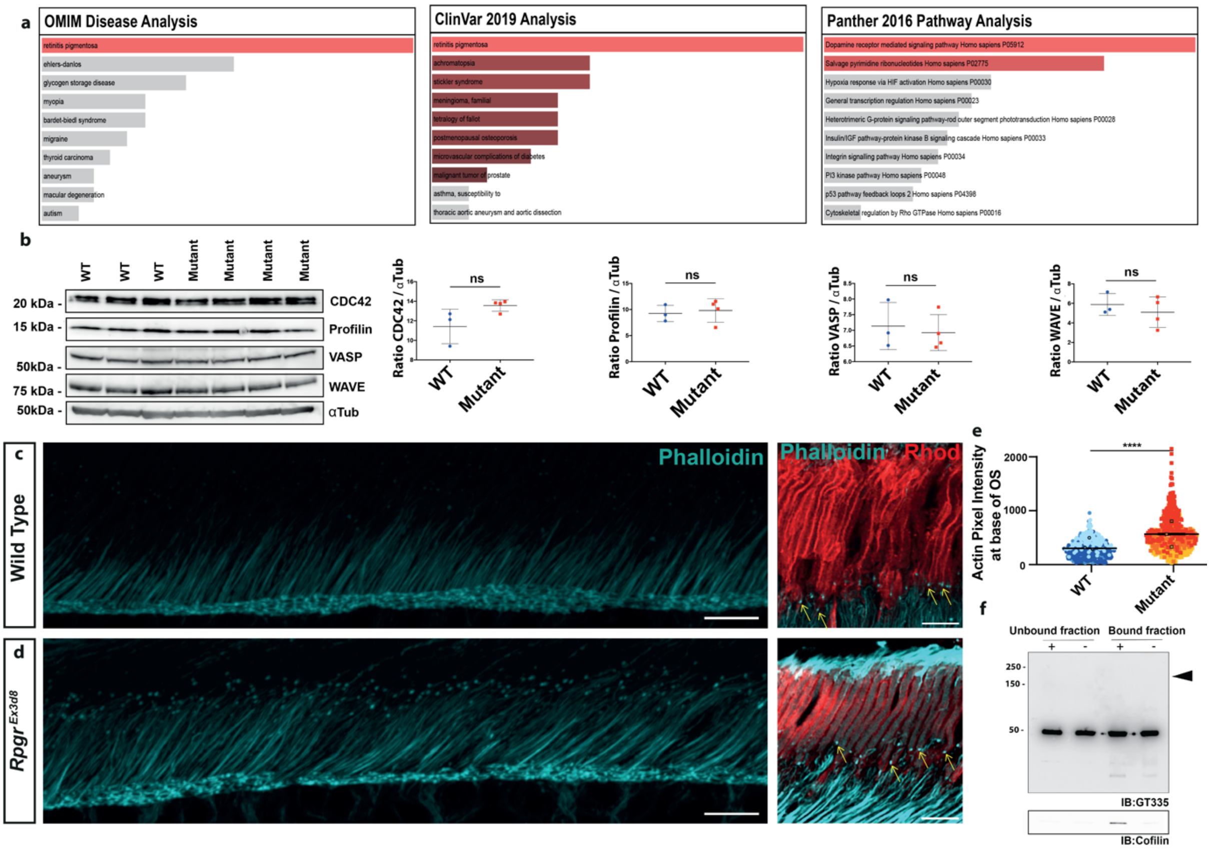

Supplementary Figure 7. The *Rpgr*^{Ex3d8} mouse has actin dysregulation in the connecting cilium (CC). (a) Differentially expressed proteins using ClinVar 2019 and OMIM disease libraries ranks retinitis pigmentosa as the top disease in *Rpgr*^{Ex3d8} retina compared to wild type control. Comparison of differentially expressed proteins to Panther 2016 protein pathway database ranked cytoskeletal regulation by Rho GTPases as a significantly disrupted pathway. **(b)** Immunoblotting of actin binding proteins and nucleators show no significant difference in protein levels in *Rpgr*^{Ex3d8} retina lysates compared to wildtype (y axis denotes ratio of protein of interest to α-tubulin loading control; experiments were performed once with statistics performed on N = 3 wild type animals and N = 4 *Rpgr*^{Ex3d8} animals; two-tailed, unpaired t-test; data are presented as mean values +/- SEM). (**c, d**) Airyscan comparison of wild type (**c**; top panels) and *Rpgr*^{Ex3d8} (**d**; bottom panels) retina show increased actin polymerisation in the distal photoreceptor CC in *Rpgr*^{Ex3d8} mice, as labelled by phalloidin. Right panels, higher magnification of actin puncta (yellow arrows) within the CC, at the base of the rhodopsin-positive outer segment. **(e)** Quantification of sum pixel intensity of photoreceptor CC phalloidin staining (N = 3 animals per genotype; **** , $p \le 0.0001$) (Scale bars = 5 μm). **(f)** Immunoprecipitation of *Rpgr*^{Ex3d8} retinal lysates using magnetic beads coated (+) or uncoated (-) with cofilin antibody shows enrichment of cofilin in bound fraction (bottom panel) and loss of detection of the retinal specific isoform of RPGR (**Figure 4d**), as detected using GT335 antibody (black arrowhead, top panel; band at 52 kDa is acetylated alpha tubulin, bands at 50 kDa and 25 kDa are immunoglobulins; experiment was performed once). Source data are provided as a Source Data file.

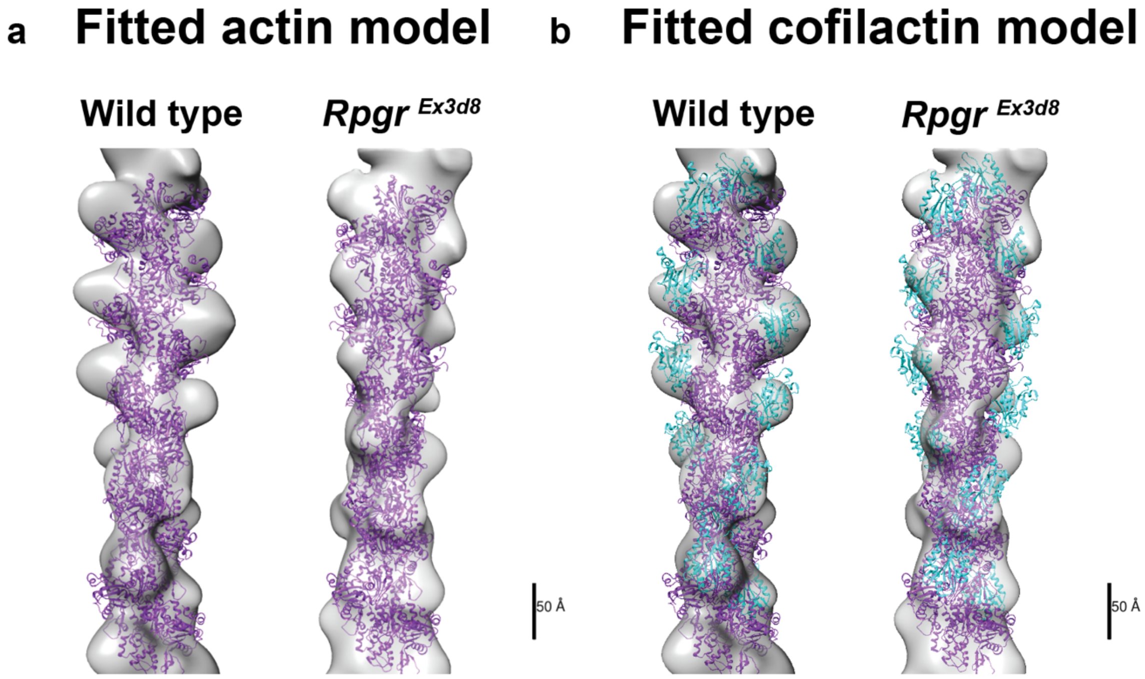

Supplementary Figure 8. Actin filament structure differs in *$Rpgr^{Ex3d8}$* distal rod connecting cilium. (A) Subtomogram averaged maps (after imposing helical symmetry) of actin from wild type and *$Rpgr^{Ex3d8}$* distal connecting cilium with fitted F-actin model (3J0S pdb, actin in magenta). **(B)** Subtomogram average maps (after imposing helical symmetry) of actin from wild type and $Rpgr^{Ex3d8}$ distal connecting cilium with fitted cofilactin model (3J0S pdb, actin in magenta, cofilin in cyan).

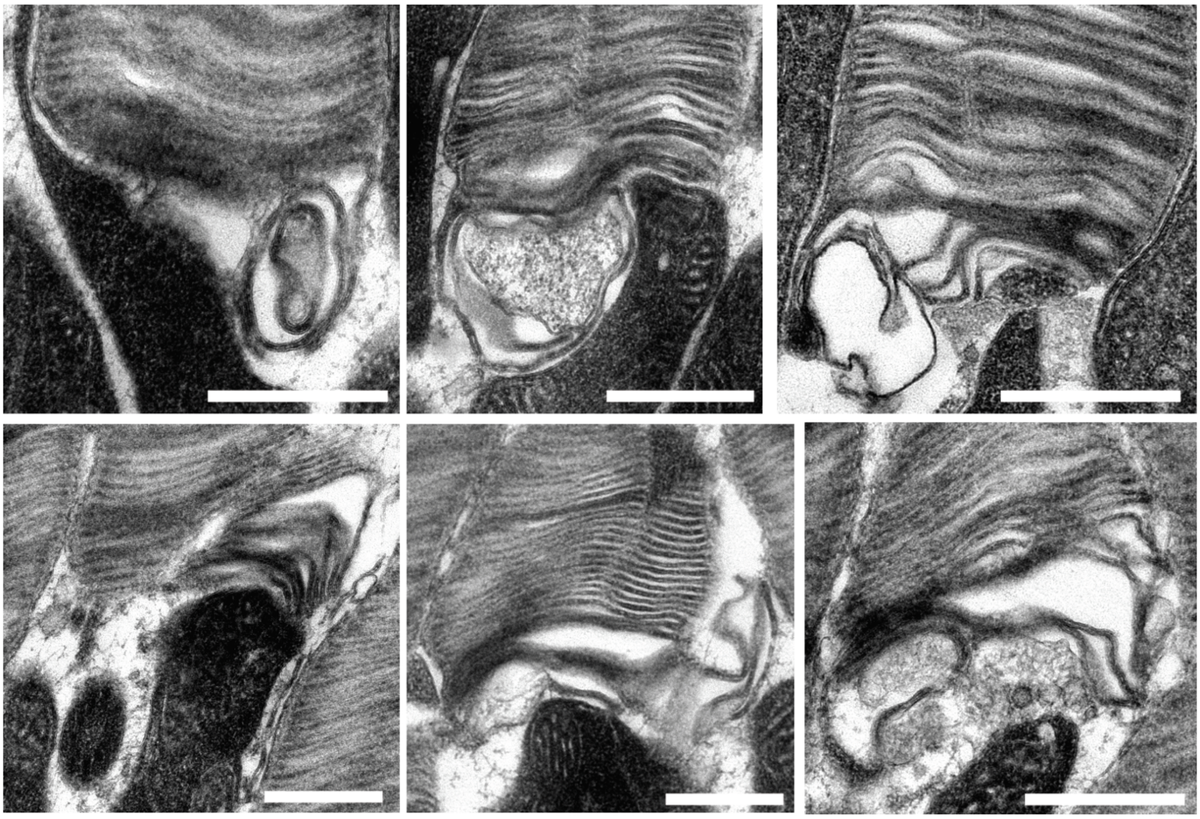

Supplementary Figure 9. Intravitreal cytochalasin D treatment results in overgrowth of photoreceptor basal discs in wild type mice. 0.5 μl of 25mM Cytochalasin D in PBS was injected intravitreally and eyes fixed after 6 hours. Images are representative of N = 3 mice (scale bars = 0.5 μm).

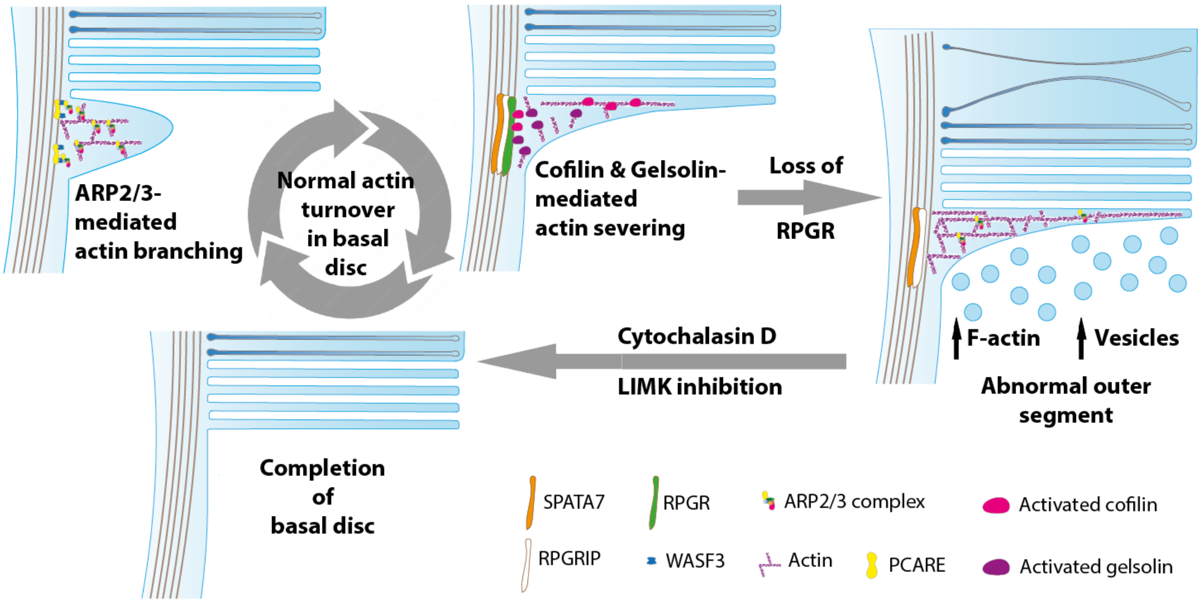

Supplementary Figure 10. RPGR is essential for controlling outer segment disc integrity. Photoreceptor disc formation is an active, actin-driven process. PCARE and WASF3 mediate ARP2/3-driven actin filament polymerisation, resulting in cilia membrane deformation and disc initiation. Membrane addition leads to disc elongation until SPATA7, RPGRIP and RPGR regulate gelsolin- and cofilin-mediated actin severing, allowing disc formation to complete. Loss of RPGR perturbs this actin severing, with abnormal discs instead being aborted and shed as ectosome-like vesicles. The resulting slowed rate of disc formation causes retinal stress, photoreceptor degeneration and loss of vision.

Supplementary Table 1. Primary antibody list				
Name	Vendor	Catalogue number	Concentration	Use
ARP2	Cell Signal Technology	3128 (RRID:AB_2181763)	1:1000	Western blot
α tubulin	Sigma	T5168 (RRID:AB_477579)	1:10000	Western blot
CDC42	Cell Signal Technology	4651 (RRID AB_10612265)	1:1000	Western blot
Cofilin	Cell Signal Technology	5175 (RRID:AB_10622000)	1:1000<br>1:250	Western blot<br>Immunohistochemistry
Phospho-cofilin (Ser 3)	Cell Signal Technology	3313 (RRID:AB_2080597)	1:1000	Western blot
Glial Fibrillary Acidic Protein	Abcam	ab7260 (RRID:AB_305808)	1:400	Immunohistochemistry
M/L-Opsin	Millipore	AB5405 (RRID:AB_177456)	1:500	Immunohistochemistry
N-WASP	Cell Signal Technology	4848 (RRID:AB_10694415)	1:1000	Western blot
Polyglutamylation Modification (GT335)	Adipogene	AG-20B-0020-C100 (RRID:AB_2490211)	1:500	Western blot
PRCD	In-house generation; Arshavsky lab		1:10000	Western blot
Profilin-1	Cell Signal Technology	3246 (RRID:AB_2163185)	1:1000	Western blot
RetP1	Chemicon	MAB5326 (RRID:AB_2156055)	1:1000	Immunohistochemistry
Rhodopsin	Millipore	MAB5356 (RRID:AB_2178961)	1:500	Immunohistochemistry
RPGR (ORF15 isoform)	In-house generation; Khanna lab			Immunohistochemistry
WAVE-2	Cell Signal Technology	3659 (RRID:AB_2216981)	1:1000	Western blot

**Supplementary Table 2.** Secondary antibody list used			
**Name**	**Vendor**	**Catalogue number**	**Concentration**
Donkey anti-mouse-Alexa488	Life Technologies	A21202	1:1000
Donkey anti-mouse-Alexa568	Life Technologies	A10037	1:1000
Donkey anti-goat-Alexa488	Life Technologies	A21432	1:1000
Donkey anti-goat-Alexa555	Life Technologies	A11055	1:1000
Donkey anti-rabbit-Alexa488	Life Technologies	A10042	1:1000
Donkey anti-rabbit-Alexa568	Life Technologies	A21206	1:1000
Horseradish peroxidase anti-rabbit	GE Healthcare	RPN4301	1:7500
Horseradish peroxidase anti-mouse	Cell Signal Technology	7076S	1:5000

**Supplementary Table 3.** List of guides, repair templates, PCR primers for generation/genotyping of novel mice strains	
**Rpgr Ex3d8 Guide**	CATGTTCGGCAGTAACAACTGGG
**Rpgr ORFd5 Guide**	GGAAGGGAAGGCAAGAAGATGGG
**Rpgr ORFd5 Repair**	GATCAAAGTAGGGAGGATGAAGGAGATAGGCAAGAGAAAGAAGGGAGAAGGGAGGGGAAGGGAAGGCAAGAAGATGGAAGGGTGG AAGGAAGGAGAGGAGCAAGAACAGGAGGAGGAAATAGAGGAAGGTGAAGAGGAAGAGAGGGAAGGAGAGGAAGAAGGGGGAGAG GAAGAGGGGGA
**Rpgr Ex3d8 detection primers**	Fd AGAACTCTCCACCGCCTACT<br>Rv AGGTGAAAAGCCTCTTGAATCA
**Rpgr ORFd5 detection primers**	Fd AGGGCTTACAAAGCAAGCAAGTAGG<br>Rv TCCTCCACATCATCCTCACAACTTC
**Floxed cofilin detection primers**	Fd1 CGCTGGACCAGAGCACGCGGCATC<br>Rv1 CTGGAAGGGTTGTTACAACCCTGG<br>Fd2 CATGAAGGTTCGCAAGTCCTCAAC
**Rhodopsin-iCre detection primers**	Fd GGGTCAGTGCCTGGAGTTG<br>Rv CAG CAT CCA CAT TCT CCT TTC<br>Rho-iCre internal positive control Fd CTAGGCCACAGAATTGAAAGATCT<br>Rho-iCre internal positive control Rv GTAGGTGGAAATTCTAGCATCATCC
