# Supplementary material for: Ciliary tip actin dynamics regulate photoreceptor outer segment integrity
Source: Nat Commun. 2024 May 21;15:4316. doi: 10.1038/s41467-024-48639-w (PMC11109262; doi:10.1038/s41467-024-48639-w)
Supplement: Supplementary file 3 — Description of Additional Supplementary Files [file 41467_2024_48639_MOESM3_ESM.pdf]

**Description of Additional Supplementary Files**

**File Name: Supplementary Data 1**
**Description: Mass spectrometry of 3 month old *Rpgr$^{Ex3d8}$* and wild type retina.**

**File Name: Supplementary Data 2**
**Description: Mass spectrometry of 6 week old *Rpgr$^{Ex3d8}$* and wild type outer segments.**

**File Name: Supplementary Movie 1**
**Description: Movie of rotating 3D tomogram of a wild type photoreceptor connecting cilium at the level of the basal disc.** Segmented reconstruction is overlaid to highlight the ciliary and disc membranes (green), microtubule-based axoneme (cyan) and microfilaments (purple). Microfilaments extend from the connecting cilium into the basal disc. Slice from this tomogram is displayed in Fig. 1b & c.

**File Name: Supplementary Movie 2**
**Description: Movie of tomographic slices along the y axis of a 3D reconstruction of a wild type photoreceptor connecting cilium at the level of the basal disc.** Slice from this tomogram is displayed in Fig. 5a.

**File Name: Supplementary Movie 3**
**Description: Movie of tomographic slices along the y axis of a 3D reconstruction of a *Rpgr$^{Ex3d8}$* photoreceptor connecting cilium at the level of the basal disc.** Slice from this tomogram is displayed in Fig 5b.

**File Name: Supplementary Movie 4**
**Description: Movie of rotating 3D tomogram of a wild type photoreceptor connecting cilium at the level of the basal disc.** Reconstruction is overlaid to highlight the ciliary and disc membranes (cyan), microtubule based axoneme (tan) and actin filaments (purple). Slice from this tomogram is displayed in Fig. 5a.

**File Name: Supplementary Movie 5**
**Description: Movie of rotating 3D tomogram of a *Rpgr$^{Ex3d8}$* photoreceptor connecting cilium at the level of the basal disc.** Reconstruction is overlaid to highlight the ciliary and disc membranes (cyan), microtubule based axoneme (tan) and actin filaments (purple). Slice from this tomogram is displayed in Fig. 5b.

**File Name: Supplementary Movie 6**

**Description: Subtomogram averaged maps (after imposing helical symmetry) of actin from wild type (left with fitted cofilactin model (3J0S pdb)) and *Rpgr*$^{Ex3d8}$ (right, with fitted F-actin model (3J0S pdb)) connecting cilia.**
