# Supplementary material for: Ciliary tip actin dynamics regulate photoreceptor outer segment integrity
Source: Nat Commun. 2024 May 21;15:4316. doi: 10.1038/s41467-024-48639-w (PMC11109262; doi:10.1038/s41467-024-48639-w)
Supplement: Supplementary file 12 — Reporting Summary [file 41467_2024_48639_MOESM12_ESM.pdf]

nature portfolio

Corresponding author(s): Roly Megaw

Last updated by author(s): 24/4/24

Reporting Summary

Nature Portfolio wishes to improve the reproducibility of the work that we publish. This form provides structure for consistency and transparency in reporting. For further information on Nature Portfolio policies, see our Editorial Policies and the Editorial Policy Checklist.

Statistics

For all statistical analyses, confirm that the following items are present in the figure legend, table legend, main text, or Methods section.

n/a	Confirmed	
☐	☒	The exact sample size (*n*) for each experimental group/condition, given as a discrete number and unit of measurement
☐	☒	A statement on whether measurements were taken from distinct samples or whether the same sample was measured repeatedly
☐	☒	The statistical test(s) used AND whether they are one- or two-sided<br>*Only common tests should be described solely by name; describe more complex techniques in the Methods section.*
☒	☐	A description of all covariates tested
☒	☐	A description of any assumptions or corrections, such as tests of normality and adjustment for multiple comparisons
☐	☒	A full description of the statistical parameters including central tendency (e.g. means) or other basic estimates (e.g. regression coefficient) AND variation (e.g. standard deviation) or associated estimates of uncertainty (e.g. confidence intervals)
☐	☒	For null hypothesis testing, the test statistic (e.g. F, t, r) with confidence intervals, effect sizes, degrees of freedom and P value noted<br>*Give P values as exact values whenever suitable.*
☒	☐	For Bayesian analysis, information on the choice of priors and Markov chain Monte Carlo settings
☒	☐	For hierarchical and complex designs, identification of the appropriate level for tests and full reporting of outcomes
☒	☐	Estimates of effect sizes (e.g. Cohen's *d*, Pearson's *r*), indicating how they were calculated

Our web collection on statistics for biologists contains articles on many of the points above.

Software and code

Policy information about availability of computer code

Data collection	Description of all code used to collect data is described in the relevant sections of the methods with references.
Data analysis	Description of all code used to analyze data is described in the relevant sections of the methods with references. All statistical analyses used are described in the figure legends along with N numbers for biological replicates for events and P values. Raw data for biological and experimental replicates has been plotted, where possible. Statistical methods used for analysis are mentioned in each figure as explained in Methods.

For manuscripts utilizing custom algorithms or software that are central to the research but not yet described in published literature, software must be made available to editors and reviewers. We strongly encourage code deposition in a community repository (e.g. GitHub). See the Nature Portfolio guidelines for submitting code & software for further information.

Data

Policy information about availability of data

All manuscripts must include a data availability statement. This statement should provide the following information, where applicable:

- Accession codes, unique identifiers, or web links for publicly available datasets
- A description of any restrictions on data availability
- For clinical datasets or third party data, please ensure that the statement adheres to our policy

Datasets from this project will be publicly available upon acceptance. The total proteomic data has been uploaded to ProteomeXchange. Mouse lines generated within this study are in the process of being given MGI terms and will shared upon acceptance.

Human research participants

Policy information about studies involving human research participants and Sex and Gender in Research.

Reporting on sex and gender	N/A
Population characteristics	N/A
Recruitment	N/A
Ethics oversight	N/A

Note that full information on the approval of the study protocol must also be provided in the manuscript.

Field-specific reporting

Please select the one below that is the best fit for your research. If you are not sure, read the appropriate sections before making your selection.

[x] Life sciences   [ ] Behavioural & social sciences   [ ] Ecological, evolutionary & environmental sciences

For a reference copy of the document with all sections, see nature.com/documents/nr-reporting-summary-flat.pdf

Life sciences study design

All studies must disclose on these points even when the disclosure is negative.

Sample size	For rescue experiments, the number of mice in experimental groups was calculated based on mice having 100% penetrance of phenotype. We used a small cohen's d effect and the standard deviation of untreated RpgrEx3d8 vesicle counts to calculate our desired effect size, used alongside an alpha value of 0.05 and a beta value of 0.20 (ie Power of 80%). We therefore ensured a minimum of 3 mice per experimental group.
Data exclusions	All raw data is plotted within graphs- there was no exclusion of individual samples or data points. All data sets of proteomics used in this study will be publicly available upon acceptance of this paper. Total proteomic data are available via ProteomeXchange with identifier PXD038015 To access the full dataset, reviewers should use the username: reviewer_pxd038015@ebi.ac.uk and password: LvDsT2Jc
Replication	Replicate information, including the number of experiments, biological and technical replicates, is provided in the figure legends. We use biological replicate to indicate an independent animal. We refer to repeat measurements of the same animal as a technical replicate. The number of data-points per condition is indicated in each sample. The means of the technical replicates for each biological replicate are indicated as a separate symbol on each graph.
Randomization	Experimental groups were assigned based on animal genotype. For image analysis, care was taken to ensure images were taken at random by choosing fields of view based on DAPI, or a stain that was not subsequently being analyzed. In most cases, images were analyzed blinded to genotype for image analysis, and if possible, image analysis was automated to reduce bias.
Blinding	Where possible, all imaging samples were acquired blinded and analysed.

Reporting for specific materials, systems and methods

We require information from authors about some types of materials, experimental systems and methods used in many studies. Here, indicate whether each material, system or method listed is relevant to your study. If you are not sure if a list item applies to your research, read the appropriate section before selecting a response.

Materials & experimental systems

n/a	Involved in the study	
☐	☒	Antibodies
☒	☐	Eukaryotic cell lines
☒	☐	Palaeontology and archaeology
☐	☒	Animals and other organisms
☒	☐	Clinical data
☒	☐	Dual use research of concern

Methods

n/a	Involved in the study	
☒	☐	ChIP-seq
☒	☐	Flow cytometry
☒	☐	MRI-based neuroimaging

Antibodies

Antibodies used	We have provided all of this data (name, catalogue number, clone number, species, fixation, dilution, distributor) in a table; Supplementary Tables 1 and 2
Validation	Antibodies used were commercially validated and use in previous publications tracked by RRIDs (see supplementary table 1) other than the PRCD antibody (gift from Arshavsky lab) and the RPGR antibody (gift from Khanna lab).

Animals and other research organisms

Policy information about studies involving animals; ARRIVE guidelines recommended for reporting animal research, and Sex and Gender in Research

Laboratory animals	All details of strain, genotype, and age are detailed in the methods and in figure legends. We studied male mice within these studies.
Wild animals	No wild animals were used in this study.
Reporting on sex	Male mice were used in this study
Field-collected samples	No field related samples were used in this study.
Ethics oversight	As stated in the manuscript methods, 'All experiments followed international, national and institutional guidelines for the care and use of animals. Animal experiments were carried out under UK Home Office Project License PPL P1914806F in facilities at the University of Edinburgh (PEL 719 60/2605) and were approved by the University of Edinburgh animal welfare and ethical review body.'

Note that full information on the approval of the study protocol must also be provided in the manuscript.
